# A new combination of a prebiotic and postbiotic mitigates immunosenescence in vaccinated healthy senior dogs

**DOI:** 10.3389/fvets.2024.1392985

**Published:** 2024-11-20

**Authors:** Wendy A. Wambacq, Emmanuelle Apper, Cindy Le Bourgot, Florence Barbe, Yang Lyu, Michael Pelst, Bart J. G. Broeckx, Bert Devriendt, Eric Cox, Myriam Hesta

**Affiliations:** ^1^Equine and Companion Animal Nutrition, Department of Morphology, Imaging, Orthopedics, Rehabilitation and Nutrition, Faculty of Veterinary Medicine, Ghent University, Merelbeke, Belgium; ^2^Lallemand Animal Nutrition, Blagnac, France; ^3^Scientific and Regulatory Affairs Department, Tereos, Moussy-le-Vieux, France; ^4^Laboratory of Immunology, Department of Translational Physiology, Infectiology and Public Health, Faculty of Veterinary Medicine, Ghent University, Merelbeke, Belgium; ^5^Department of Veterinary and Biosciences, Faculty of Veterinary Medicine, Ghent University, Merelbeke, Belgium

**Keywords:** scFOS, prebiotic, postbiotic, immunosenescence, elderly dog, vaccine

## Abstract

**Introduction:**

Elderly dogs often suffer from chronic diseases, in part attributed to immunosenescence, characterized by reduced blood CD4+ T cells (helper T cells) and elevation in the CD8+ T cells subset (cytotoxic T cells). Studies conducted in adult dogs suggested that supplementing short chain fructo-oligosaccharides (scFOS) or postbiotics derived from yeasts may positively influence the immune response. The aim of the current study was to investigate whether dietary supplementation with a combination of scFOS with a new yeast postbiotic (Profeed ADVANCED^®^ called scFOS+) could have a positive influence on the immune status of senior dogs subjected to an immune challenge (Lyme disease vaccination).

**Methods:**

To this end, 22 healthy senior client-owned dogs were divided into two groups: one group received a placebo diet without scFOS+ and the other group the basal diet supplemented with 1.1% scFOS+. In order to assess immune function, complete blood count, serum acute phase proteins, immunoglobulins, cytokines, T-cell subsets and antibody secreting cells were analyzed. Furthermore, faecal score and pH were recorded.

**Results and discussion:**

Dogs fed the scFOS+ supplement had decreased total serum IgA concentrations (*p* < 0.01), which might suggest a more local IgA response in agreement with what was previously found when adult dogs were supplemented with *β*-1, 3/1, 6-glucan, a yeast-based product. More importantly, the present study demonstrated that feeding 1.1% scFOS+ to healthy senior dogs increased the CD4+:CD8+ T-cell (Helper:Cytotoxic T cell) ratio (*p* < 0.001) during and after vaccination against Lyme disease. Combining scFOS and yeast-derived postbiotics in the diet can therefore counter certain characteristics of T-cell immunosenescence in dogs.

## Introduction

The increased interest in providing adequate care for senior pets (1, 2) as well as the improvements in veterinary medicine over recent decades have resulted in a growing senior dog population (3). Thirty to 40% of all dogs with complaints presented to the vet today are senior animals with age-related specific needs (4). Once a dog has reached senior age, a decline in physical condition, organ function and immune response occurs (5). Immunosenescence, defined as the decline of immune function with age, is well-characterized in humans and has been reported in dogs. Indeed, older dogs respond less effectively to a primary immunization (cf. primo vaccination). More specifically, the induced antibody titers to novel antigens were reduced in senior animals as compared to younger dogs (6). This decreased immune function in senior dogs increases their susceptibility to infectious diseases. In humans, immunosenescence is hallmarked by pronounced changes in the T cell population like, among other events, a decrease in blood CD4+ T cells and an increase in blood CD8+ T cells (7). In senior dogs and cats, reduced blood CD4+ T cells have also been reported, while the evolution of CD8+ remains unclear and inconsistent (3). A previously conducted study using flow cytometry revealed that the naïve population of both CD4+ and CD8+ T cells decreased with age, while CD4+ and CD8+ memory T cells displayed more cytokine production (8). A recent study also indicated that T-lymphocyte ratio (CD4+/CD8+) was significantly correlated with age in dogs (9). Since immune protection for emerging infectious diseases, such as Lyme disease, remains desirable for these animals, it is thus of interest to find strategies to alleviate the negative impact of immunosenescence.

Immunonutrition is defined as the potential to modulate the activity of the immune system by interventions with specific nutrients (10). The immune system can be modulated in a direct matter, or it can be affected indirectly through gut microbiota modulation. In this respect, pre-, pro- and postbiotics represent good candidates to develop nutritional strategies as they are able to exert a crosstalk with the host’s cells and its microbiota. The use of prebiotics may be beneficial for the elderly population (11). Short chain fructo-oligosaccharides (scFOS) are fermentable fibers that fall into the group of prebiotics. Prebiotics are fermented by beneficial microbiota present in the large intestine, which allows it to thrive and repress pathogens (12). In addition, these prebiotic fibers may also positively influence the local and systemic immune response in dogs, through their interaction with the gut-associated lymphoid tissue (13). Beside the prebiotic fibers, the use of yeast fractions has been reported as a positive nutritional strategy to support immunity. As an example, immune benefits have been observed when mannan oligosaccharides (MOS) from *S. cerevisiae* were used in combination with scFOS in adult healthy dogs (11). Yeast fractions can be provided by several types of yeasts, including not only *Saccharomyces* but also non-*Saccharomyces*, and, depending on their composition, can be considered as postbiotics, i.e., “a preparation of inanimate microorganisms and/or their components that confers a health benefit on the host” (14). An innovative and patented combination of different types of yeast fractions together with scFOS could be a good nutritional strategy to mitigate immunosenescence of old dogs. Therefore, the aim of the current study was to investigate whether dietary supplementation with the prebiotic scFOS combined with specifically selected yeast fractions (Profeed ADVANCED^®^ product: scFOS+) could have a positive influence on the immune status within this ever-growing senior dog population. This is, to the authors’ knowledge, the first study utilizing a vaccine response (Lyme disease vaccine) as a parameter for assessing both humoral and cellular immune function in a senior dog population fed this combination.

## Materials and methods

### Experimental design

The research protocol was evaluated and approved by the Ethical Committee of the Faculty of Veterinary Medicine, Ghent University, Belgium (EC 2017/103) and was in accordance with institutional and national guidelines for the care and use of animals. A randomized, placebo-controlled double blinded *in vivo* experiment was conducted between March 2018 and June 2019, with dog owners being able to enter the study with their senior dog at any given time point. Senior dogs were included into the cohort according to the criteria defined in the “Animals” section and were divided into 2 groups: placebo and scFOS+ groups.

A previously published human/pet age analogy chart (15) was used to determine whether a dog could be considered senior. This chart accounted for the fact that small dog breeds live longer than larger breed dogs.

### Animals

**Figure 1:**
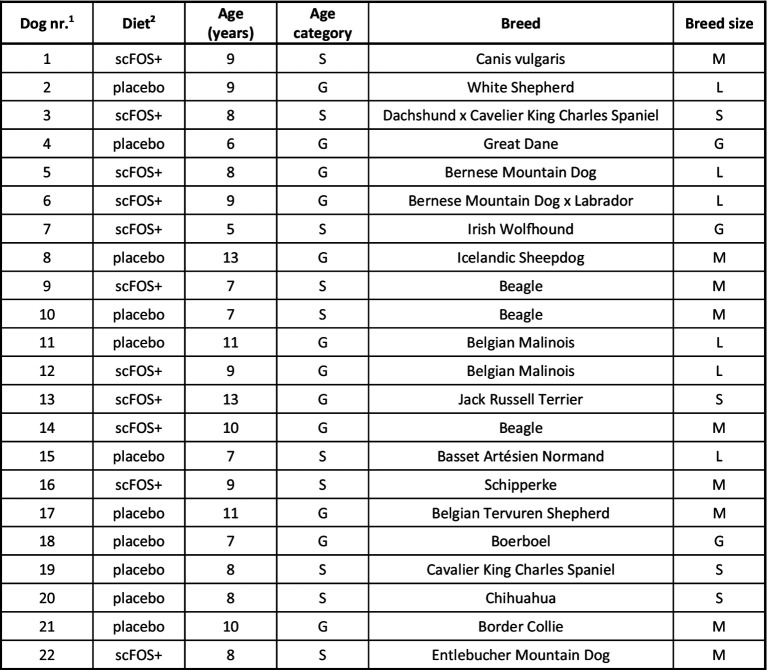
Diet, age and breed of dogs included in the study. S, senior; G, geriatric; S, small breed; M, medium breed; L, large breed; G, giant breed. ^1^Order in which the dogs entered the study. ^2^Type of diet received by each dog: placebo diet or scFOS+ diet supplemented with Profeed ADVANCED^®^.

A total of 22 healthy senior client-owned dogs (Figure 1) were included in the study and homogeneously distributed by sex, age, body weight (BW) and body condition score (BCS) between the two groups.

A thorough health screening was performed prior to the study (day 0, D0). This included a clinical examination, urine analysis (Siemens Multistix^®^ 5 dipstick) and analysis of a fasting blood sample (complete blood count and biochemistry including urea, creatinine, total protein, aspartate transaminase, alanine transaminase, gamma-glutamyl transferase, alkaline phosphatase and glucose).

Inclusion criteria used for study participation were that the dogs should not be receiving any medication and should be familiar with eating kibbles. They should appear healthy on general clinical examination (the dogs had to be dewormed—Caniquantel Plus, Fendigo sa/nv, Brussels, Belgium—3 days prior), have normal fasting blood outcomes and absence of protein in the urine. Unfortunately, there are no established age-dependent laboratory reference intervals for older dogs-selecting an appropriate reference population is a major challenge, as older dogs might suffer from subclinical disease- (16). As a result, a consultation with an internal medicine specialist (ECVIM diplomate) was arranged to review and discuss any results exceeding the standard laboratory reference intervals. Only senior dogs with laboratory abnormalities deemed insignificant were allowed to participate in the study. The BCS had to be between 3 and 6 out of 9 (17). The dogs should have received their core vaccines (against canine distemper virus, adenovirus and parvovirus type 2) at an early stage of their life. Finally, they should never have received a vaccine against Lyme disease (*Borrelia burgdorferi*) prior to the study, as demonstrated with a negative Canine Lyme Antibody Rapid Test (Abaxis Europe GmbH, Griesheim, Germany).

### Diets and vaccination

Two diets were formulated to meet the National Research Council requirements for adult dogs (18). Both diets contained dehydrated chicken (28%), rice, rice flour, animal fat, cellulose (2%), beet pulp (1%), brewer’s yeast, minerals, dried whole eggs and lecithin. The scFOS+ diet, unlike the placebo diet, was supplemented with 1.1% Profeed ADVANCED^®^, a compound feed composed of scFOS prebiotic fibers combined with a new yeast postbiotic (Beghin-Meiji; France), added before extrusion. Water always remained available.

The experimental diets were subjected to Weende (proximate) analysis. They were dried to a constant weight at 103°C to determine dry matter (DM, ISO 1442, 1997). Crude ash was determined by combustion at 550°C (ISO 936, 1998). Crude protein was calculated from Kjeldahl nitrogen (6.25× N, ISO 5983-1, 2005). Crude fiber was analyzed by acid-alkali digestion (ISO 5498, 1981), and crude fat was analyzed using acid-hydrolysis followed by Soxhlet extraction (ISO 1443, 1973) (Table 1).

**Table 1:** Composition of the dry extruded dog diets.

	Placebo diet^1^	scFOS+ and diet
Crude protein (% on DM)	20.7 (20.7)^2^	22.4 (20.5)
Crude fat (% on DM)	7.2 (8.9)	7.1 (8.9)
Crude fiber (% on DM)	2.0 (1.7)	2.6 (1.7)
Crude ash (% on DM)	5.8 (7.2)	6.1 (7.1)
NFE^3^ (% on DM)	64.3 (61.6)	61.9 (61.8)
ME^4^ (MJ/100 g DM)	1.600 (1.622)	1.582 (1.623)

DM, dry matter; NFE, nitrogen-free extract; ME, metabolizable energy.

1 Both diets contained dehydrated chicken (28%), rice, rice flour, animal fat, cellulose (2%), beet pulp (1%), brewer’s yeast, minerals, dried whole eggs and lecithin.

2 First batch (second batch).

3 NFE (% on DM) was calculated as 100 – crude protein – crude fat – crude fiber – crude ash, with all components on DM basis.

4 ME was calculated as (((5.7 × g protein) + (9.4 × g fat) + (4.1 × (g NFE + g fiber))) × (91.2 – (1.43 × % crude fiber in DM))/100) – (1.04 × g protein)/1,000 (18).

Following the health screening, dogs were accustomed to the new diets by a gradual 7-day transition period. Subsequently, all dogs were fed their respective diets for 14 consecutive weeks. The diets were fed in amounts to maintain the animal’s body weight constant throughout the study. To this end, daily maintenance energy requirements (18) were fed based on each animal’s ideal body weight, except for one dog which was fed the placebo diet that started the study with a BCS of 3/9. This dog was fed to reach a BCS of 4/9 during the first 4 weeks of the study (up until the vaccination). The body weight and food intake of all dogs were recorded, respectively, once and twice per week by the owner.

**Figure 2:**
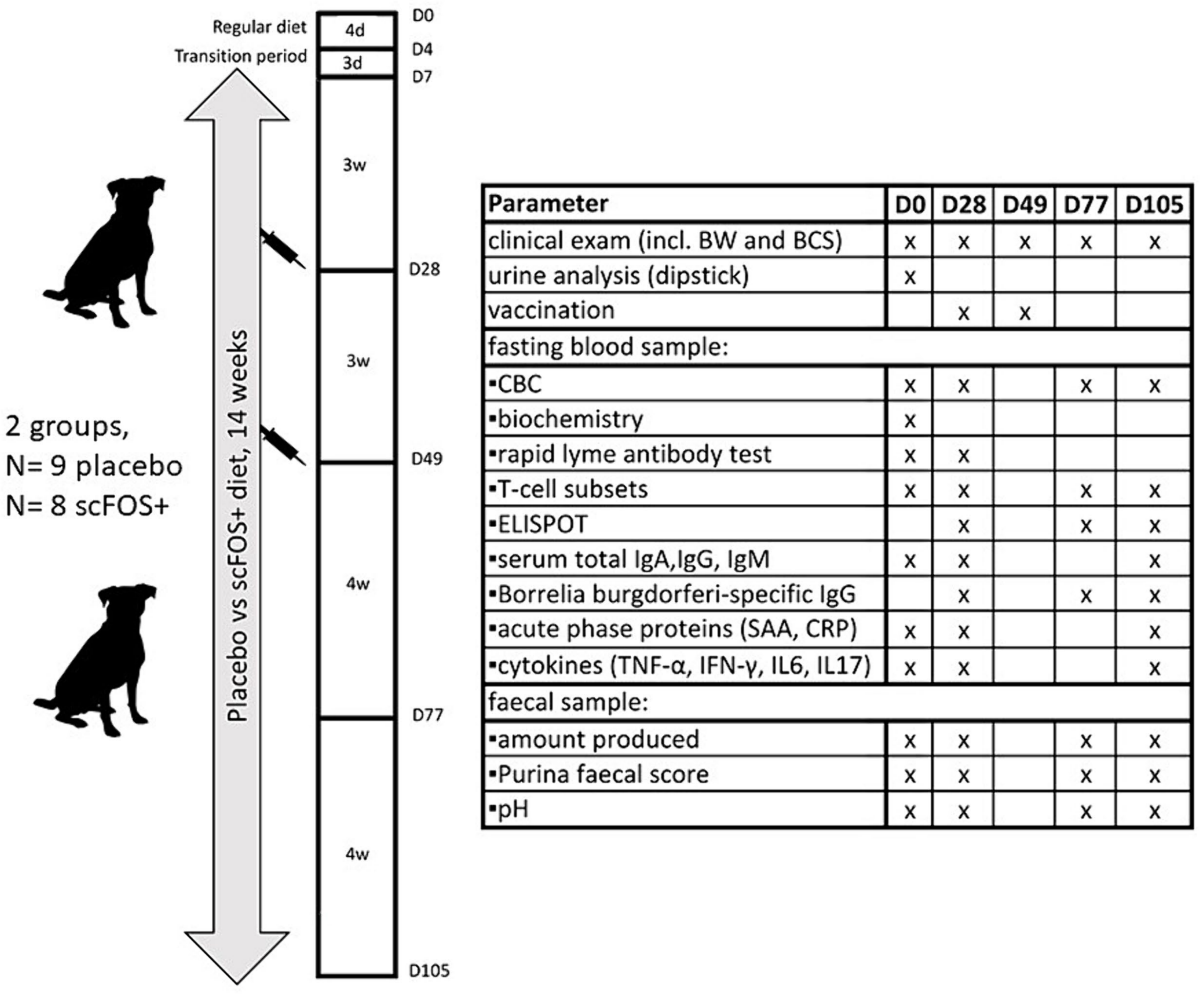
Schematic overview of the experimental design and analyzed parameters. D, day; d, days; w, weeks; BW, body weight; BCS, body condition score (1–9/9); CBC, complete blood count; SAA, serum amyloid A; CRP, C-reactive protein.

Three weeks after exclusively consuming the test diets (day 28, D28), dogs were vaccinated with a Lyme disease vaccine (Merilym-3, Merial, Diegem, Belgium, a trivalent vaccine containing inactivated *Borrelia garinii*, *Borrelia afzelii, Borrelia burgdorferi sensu stricto* and aluminium hydroxide as the adjuvant). This vaccination was repeated after 3 weeks (booster, day 49, D49), as indicated on the vaccine leaflet. Dogs continued to consume their respective test diets for 4 weeks (until day 77, D77) and then another 4 weeks (day 105, D105). Dog owners were again invited to the veterinary clinic of Ghent University for follow-up consultations at these time points (Figure 2).

### Sample collection

Blood samples were collected via jugular venipuncture (35 mL per dog) on D0 (before starting the test diets), D28 (before vaccination), D77 and D105 (4 and 8 weeks after the booster vaccination respectively), following an overnight fast. Samples were directly collected in vacutainer serum tubes for biochemistry, serum immunoglobulin, acute phase protein and cytokine analysis. Additionally, blood was collected in vacutainers containing lithium heparin for peripheral blood mononuclear cell (PBMC) isolation. Sodium fluoride vacutainers were used to measure blood glucose levels and vacutainers containing dipotassium EDTA were used to perform complete blood count (CBC) analysis and a Canine Lyme Antibody Rapid Test (Abaxis Europe GmbH, Griesheim, Germany). Serum samples were centrifuged at 2431 × *g* (5 min at 21°C) and supernatants were collected, aliquoted and frozen at −20°C (for immunoglobulins and acute phase proteins) and −80°C (for cytokines) awaiting further analysis. All other blood samples were processed further on the day of collection. Additionally, naturally voided faecal samples were collected within 15 min after defecation and immediately stored at −20°C with the respective owners within 24 h prior to the consultation at D0, D28, D77 and D105. These samples were then brought to the veterinary clinic of Ghent University using cooling elements during transport and were consequently analyzed for faecal score and pH.

### Isolation of PBMC from heparinized canine blood

Blood samples (5 mL at D0 and 10 mL at D28, D77 and D105, contained in lithium heparin Vacutainer tubes) were kept at room temperature (RT, 18–22°C) until processing. Within 4 h after sample collection, heparinized blood samples were diluted 1/1 with sterile phosphate buffered saline (PBS). Isolation of PBMC was achieved using a Ficoll density gradient centrifugation at 900 g for 30 min at 18°C [7.1% Ficoll PM400 and 9% sodium diatrizoate hydrate (Merck, Burlington, MA, USA)] (19). The interphase containing the PBMC was collected, and cells were washed in an equal volume of Alsever’s solution (pH of 6.1, VWR, Radnor, PA, USA; centrifugation at 300 *g* for 10 min at 18°C), after which the supernatant was removed. Erythrolysis was performed using a lysis buffer [140 mM NH_4_Cl, 17 mM Tris (VWR), with a pH of 7.2] for 5 min at RT. Then, cells were washed again with 5 mL Alsever’s solution for 10 min at 400 g.

### Phenotyping lymphocyte subpopulations by flow cytometry

Subpopulations of T-lymphocytes (CD4+, CD8+) were analyzed using flow cytometry [test validation was performed according to the protocol described by Selliah et al. and Maina et al. (20, 21)]. To this end, isolated PBMC were resuspended in PBS and brought on a 96-well V-bottom plate at 5 × 10^5^ cells/well. Subsequently, 50 μL of 1 in 8 diluted CD3/CD4/CD8 antibodies (anti-Dog CD3:FITC/CD4:RPE/CD8:Alexa Fluor^®^ 647, Bio-rad, Veenendaal, The Netherlands) was added. Plates were incubated and kept on ice (dark environment) for 20 min. Hereafter, cells were washed twice in PBS + 1% bovine serum albumin (BSA) for 3 min at 4°C and 400 g. The proportion of lymphocyte subpopulations was determined by flow cytometry (CytoFlex, Beckman Coulter, Life Sciences, Woerden, Netherlands) using the CytExpert 2.0 software (Beckman Coulter), with a minimum event count of 20,000 for each sample.

### Total IgA and *Borrelia burgdorferi*-specific IgA antibody secreting cells (ASC) ELISPOT

The ELISPOT tests were performed according to a protocol developed by Pelst et al. (22), with some modifications. For each individual dog, three sets of three wells of a Polysorp 96-well plate (Thermofisher Scientific) were coated with solely bicarbonate buffer (pH 9.4), bicarbonate buffer containing 10 μg/mL native *Borrelia burgdorferi* antigen (EastCoast bio, North Berwick, USA) and 5 μg/mL goat anti-canine IgA (Bethyl Laboratories) in bicarbonate buffer, respectively. After coating for 16 h at RT, the plate was blocked for another 2 h using bicarbonate buffer with 2% gelatin from cold water fish skin (Merck). After washing [PBS + 0.05% Tween^®^20 (Merck)], 50 μL of cell suspension [10×10^6^ PBMC/ml of complete RPMI (Roswell Park Memorial Institute 1,640 medium supplemented with 10% fetal calf serum, 2 mM L-glutamine), 1% MEM non-essential amino acid solution, 1 mM sodium pyruvate, 100 U/mL penicillin, 100 μg/mL streptomycin and 100 μg/mL kanamycin (Thermofisher Scientific, Waltham, MA, USA)] was added to the wells. Cell suspensions were diluted to 2×10^6^, 1×10^6^, and 5×10^5^ PBMC/ml in complete RPMI before addition to separate wells that were coated with goat anti-dog IgA (Bethyl, Montgomery, AL, USA). The cell suspensions were maintained for 16 h at 37°C and 5% CO_2_. Subsequently, the cells were removed from the plate by washing with PBS + 0.2% Tween^®^20 (Merck) and 80 ng/mL of goat anti-dog IgA horseradish peroxidase (HRP) (Bethyl Laboratories) in bicarbonate buffer +2% fish gelatin (Merck) was added for 2 h at RT. After washing [PBS + 0.05% Tween^®^20 (Merck)], 50 μL of 3,3′,5,5′-Tetramethylbenzidine (TMB) liquid substrate system for membranes (Merck) was added to each well for 5 min at RT. After aspiration of the fluid, the plate was scanned with an ImmunoSpot analyzer (Cellular Technology Limited, Cleveland, USA) and spots were counted using the autocount function of the ImmunoSpot 4.0 software (Cellular Technology Limited). Background spots were determined by the number of spots present in the wells containing the cell suspensions in uncoated but blocked wells. The average number of background spots was then subtracted from the number of spots in the respective *Borrelia burgdorferi* and anti-IgA coated wells. The total IgA-ASC were determined by multiplying the number of spots with the dilution of the cell suspension. Results were expressed as (1) the percentage of the well area that was covered with *Borrelia* IgA ASC, (2) the percentage of the well area that was covered with IgA (total) ASC, (3) the number of *Borrelia burgdorferi*-specific IgA ASC/total IgA ASC, and (4) the number of IgA ASC per 500.000 PBMC. In order to validate the coating procedure, blood plasma of a dog known to have IgA antibodies to *Borrelia burgdorferi* was used as a positive control on each plate [test validation was performed according to Janetzki et al. (23)].

### Determination of total serum immunoglobulin concentrations

The total IgA, IgG and IgM concentrations in serum were determined by a sandwich enzyme-linked immunosorbent assay (ELISA), with test validation performed according to Minic and Zivkovic (24). A Nunc Maxisorp 96-well plate (Thermofisher Scientific) was coated for 1 h (RT) with 10 μL/mL goat anti-dog IgA, IgG, or IgM (Bethyl, Montgomery, AL, USA) in bicarbonate buffer (pH 9.4), after which nonspecific binding sites were blocked with 250 μL blocking solution (0.05 M Tris-base, 0.15 M NaCl, 1% BSA, pH 8.0) for 30 min at RT. After washing five times (0.05 M Tris-base, 14 M NaCl, 0.05% Tween^®^20, pH 8.0), serial dilutions of a standard reference serum sample with known isotype-specific Ig concentrations—dog reference serum (Bioké) and dog gamma globulin (Jackson ImmunoResearch) for IgA/IgM and IgG, respectively-were added to every plate to obtain a calibration curve. Based on earlier performed serial dilutions in test samples, blood serum was diluted 1/2000, 1/512000 and 1/4800 in dilution buffer (0.05 M Tris-base, 0.15 M NaCl, 1% BSA, 0.05% Tween^®^20, pH 8.0) and added to the wells in duplicate (1 h at RT) for detection of IgA, IgG and IgM, respectively. After washing, 1/10000 HRP-conjugated goat anti-dog IgA, 1/10000 goat anti-dog IgG HRP, or 1/5000 goat anti-dog IgM HRP (Bethyl, Montgomery, AL, USA) in dilution buffer was added to the wells and the plates were incubated for 1 h at RT. Finally, after washing, 50 μL freshly prepared, 1 mg/mL 2,2-azino-di-(3-ethylbenzthiazoline sulfonate) diammonium salt (ABTS, Merck, Burlington, MA, USA) was added to each well and incubated for 15 min at 37°C. The optical density (OD) was read at 405 nm and the concentration of each immunoglobulin in a sample was calculated from the calibration curves.

### Determination of *Borrelia burgdorferi*-specific IgG in canine serum

For determination of *Borrelia burgdorferi*-specific IgG in canine serum, a quantitative ELISA was developed [test validation was performed according to Minic and Zivkovic (24)]. A Polysorp 96-well plate (Thermofisher Scientific) was coated with 10 μg/mL of native *Borrelia burgdorferi* antigen (EastCoast bio, North Berwick, USA) in bicarbonate buffer with pH 9.4. Coating occurred for 16 h at RT, followed by blocking for 30 min at RT using 0.05 M Tris-base, 0.15 M NaCl and 1% BSA at pH 8.0. Earlier, serial dilutions of the calibrator and positive control of a semi quantitative *Borrelia burgdorferi* IgG test (anti-*Borrelia* ELISA Dog IgG, EuroImmun, Lübeck, Germany) were inserted into a canine IgG ELISA (see above) to determine the amount of *Borrelia* IgG present (0.09 mg/dL and 2.3 mg/dL, for the calibrator and positive control, respectively). After washing the *Borrelia*-coated plate five times (0.05 M Tris-base, 14 M NaCl, 0.05% Tween^®^20, pH 8.0), serial dilutions of the calibrator and positive control used as standard reference samples and blood serum samples, diluted 1/800 in dilution buffer (0.05 M Tris-base, 0.15 M NaCl, 1% BSA, 0.05% Tween^®^20, pH 8.0), were added to the wells for 1 h at RT. After washing 5 times, 1/10000 HRP-conjugated goat anti-dog IgG (Bethyl, Montgomery, AL, USA) in dilution buffer was added to the wells and the plate was incubated for 1 h (RT). Subsequently, after washing, 50 μL 1 mg/mL ABTS (Merck, Burlington, MA, USA) was added to each well and incubated for 15 min at 37°C. Absorbance was read at 450 nm with a microplate reader and results were analyzed by Deltasoft JV 2.1.2. All samples were analyzed in duplicate.

### Acute phase protein analysis and ELISA to determine serum concentrations of TNF-*α*, IFN-*γ*, IL-6 and IL-17

Serum samples were analyzed for serum amyloid A (SAA, spectrophotometry, idfiSIS) and canine C-reactive protein (CRP, turbidimetry, Abbott architect C16000). Furthermore, serum samples for the analyses of TNF-*α*, IFN-*γ*, IL-6 and IL-17A were collected at D0, D28 and D105 and frozen at −80°C awaiting further analysis. These cytokines were determined (duplicate) using commercially available canine ELISA test kits (canine TNF-α ELISA, Raybiotech, Georgia, USA; canine IFN-γ ELISA, Raybiotech, Georgia, USA; canine IL-6 ELISA kit, Cloud-Clone Corp., Texas, USA; canine IL-17 ELISA kit, Wuhan Fine Biotech Co., Ltd., Wuhan, China) according to the manufacturer’s instructions.

### Faecal analyses

Faecal pH was measured in triplicates using a portable pH meter (Hanna Instruments, Temse, Belgium). Fresh faecal consistency (1: very hard and dry; 2: firm, but not hard; 3: log-like; 4: very moist; 5: very moist but has distinct shape; 6: has texture, but no defined shape; 7: watery, no texture, flat) was evaluated using Purina^®^ Faecal Scoring System (25).

### Statistical analysis

All analyses were run in R version 4.0.2 (“Taking off again”). The analysis consisted out of 3 parts. Firstly, the individual effect of four independent variables (diet, timepoint, senior/geriatric age category, and breed size—small/medium/large/giant -) on the various outcome parameters was evaluated using linear mixed models with dog as random effect. Secondly, a forward selection procedure was applied to identify the potential association of multiple independent variables (and their interactions) on these outcomes using a linear mixed model with dog as random effect. Finally, a linear mixed model with dog as random effect and diet, timepoint and their interaction as fixed effect was used to evaluate the effect of diet over time. *p*-values below 0.05 were set as statistically significant and a *p* value between 0.05 and 0.10 was defined as a trend.

## Results

### Cohort description

According to the chart we used (14), the placebo group contained 4 senior and 7 geriatric dogs, whereas the scFOS+ supplemented group consisted of 6 senior and 5 geriatric dogs. There were 2 small breed dogs, 4 medium, 3 large and 2 giant breed dogs in the placebo group, and 2 small breed dogs, 5 medium, 3 large and 1 giant breed dog in the scFOS+ supplemented group. A mean age of 8.8 (standard deviation, SD2.2) and 8.6 (SD2.0) years, a mean BW of 25.7 (SD20.9) and 25.8 (SD20.8) kg and a mean BCS of 5/9 (SD1.0) and 5/9 (SD0.7) was observed for the placebo and scFOS+ supplemented group, respectively. Whereas the placebo-supplemented group contained 7 females and 4 males, the scFOS+ group consisted of 4 female and 7 male dogs. Both groups each contained 3 spayed females and 3 spayed males.

Only 17 dogs completed the entire study. Two dogs did not reach D49 (due to a bite incident and lameness requiring NSAID administration), 2 dogs were withdrawn because they did not reach D77 (due to tonsillar and testicular cancer development), and one dog did not reach D105 due to severe acute pneumonia, highlighting the difficulty to work with a senior dog population. Data from the aforementioned dogs were included into the statistical analysis up until the end of their participation into the study.

### Chemical composition of diets, energy intake, body condition score and faecal parameters

The analyzed chemical composition of the diets is shown in Table 1. Daily metabolizable energy intakes [kJ/(kg idBW^0.75^ day)] during the stable body weight phase of the study (D28-D105) did not differ between diets (*p* = 0.341), and were not affected by time, age category and breed size, nor by the interaction of ‘diet × time.’ Body condition score remained stable for all the dogs during the entire stable body weight phase of the study (*p* = 0.980) and was not affected by diet (*p* = 0.743) nor any other variable taken into consideration. Results revealed no significant variations regarding age category, breed size, diet nor ‘diet × time’ for faecal score and faecal pH (Table 2). A significant time effect was found for faecal pH (*p* = 0.037) with a significant increase at D28 (*p* = 0.037) and a trend to increase at D105 (*p* = 0.094). Time effects are reported in Table 2.

**Table 2:** *P*-values for effect of diet, time, age category, breed size and ‘diet × time’ interaction for energy intake, body condition score and faecal parameters in dogs fed the scFOS+ and placebo diets.

	*p*-value				
	Diet	Time	Age category	Breed size	Diet × time
ME (kJ/(kg idBW^0.75^ day))^1^	0.341	0.241	0.383	0.208	0.866
BCS (1–9/9)^1^	0.743	0.980	0.527	0.644	0.898
Faecal score (1–7/7)^2^	0.770	0.595	0.668	0.111	0.520
Faecal pH	0.865	**0.037**	0.856	0.178	0.108

ME, mean metabolizable energy intake in between sample time points (D0-D28, D28-D49, D49-D77, D77-D105); idBW, ideal body weight; FM, fresh matter.

1 During the stable body weight phase of the study (D28-D105).

2 Purina faecal scoring system (1: very hard and dry; 2: firm, but not hard; 3: log-like; 4: very moist; 5: very moist but has distinct shape; 6: has texture, but no defined shape; 7: watery, no texture, flat).

Bold value means *p*-value ≤ 0.05.

### Blood parameters

All dogs have been tested negative on the Canine Lyme Antibody Rapid Test at both D0 and D28, demonstrating they were never vaccinated before, nor had they encountered Lyme disease before. All other blood parameters are shown in Table 3. A significant diet effect was found for total serum IgA (*p* = 0.006), indicating a decrease of on average 106.5 mg/dL in scFOS+ fed dogs. There were no other effects of diet nor of the interaction ‘diet × time’ found for the remaining serum immunoglobulins, nor for CBC values, cytokines, acute phase proteins and antibody secreting cells. Of note, a large inter-and intra-individual variability was observed for the tested cytokines.

**Table 3:** *P*-values for effect of diet, time, age category, breed size and ‘diet × time’ interaction for haematology (CBC), acute phase proteins, cytokines, serum immunoglobulins, T-cell subsets and ELISPOT data in dogs fed the scFOS+ and placebo diet.

	*p*-value				
	Diet	Time	Age category	Breed size	Diet × time
Leukocytes (/μL)	0.293	0.267	0.759	*0.084*	0.252
Neutrophils (/μL)	0.368	0.284	0.703	0.140	0.285
Lymphocytes (/μL)	0.323	0.706	0.916	*0.080*	0.334
Monocytes (/μL)	0.662	0.185	0.997	*0.082*	0.316
Basophils (/μL)	0.619	0.597	0.305	0.166	0.804
Eosinophils (/μL)	0.356	0.829	0.996	0.160	0.860
Neutrophils (% of total WBC)	0.804	0.194	0.956	0.886	0.464
Lymphocytes (% of total WBC)	0.712	0.315	0.933	0.291	0.312
Monocytes (% of total WBC)	0.629	0.626	0.843	0.187	0.839
Basophils (% of total WBC)	0.687	1.000	0.241	0.599	0.737
Eosinophils (% of total WBC)	0.797	0.265	0.777	*0.098*	0.991
SAA (mg/L)	0.163	0.845	0.223	0.128	0.672
CRP (mg/L)	0.321	1.000	0.273	0.218	0.290
TNF-α (pg/mL)	0.766	0.197	0.368	0.519	0.993
IFN-γ (ng/mL)	0.403	0.186	0.478	0.566	0.314
IL6 (pg/mL)	0.363	0.176	0.973	0.413	0.608
IL17A (pg/mL)	0.165	0.734	0.763	0.545	0.221
Total serum IgA (mg/dL)	**0.006**	0.332	0.914	0.497	0.778
Total serum IgG (mg/dL)	0.314	0.330	0.233	0.435	0.191
Total serum IgM (mg/dL)	0.967	**<0.001**	0.270	0.178	0.452
*Borrelia*-specific serum IgG (mg/dL)	0.891	**<0.001**	0.343	0.242	0.859
Percentage of well area covered with *Borrelia* IgA ASC (%)	0.728	0.843	0.614	0.367	0.678
Percentage of well area covered with total IgA ASC (%)	0.237	*0.058*	0.820	0.260	0.122
*Borrelia* IgA ASC/500.000 PBMC	0.311	0.907	0.849	0.359	0.616
Total IgA ASC/500.000 PBMC	0.416	**0.003**	0.714	0.328	0.765
*Borrelia* IgA ASC/total IgA ASC	0.304	0.921	0.763	0.286	0.817
CD4 + CD8-T-cells (%)	**0.022**	*0.099*	0.632	**0.008**	0.900
CD4-CD8+ T-cells (%)	*0.089*	0.173	0.481	**0.013**	0.367
CD4-CD8- (DN) T-cells (%)	0.383	0.326	0.561	0.646	**0.042**
CD4 + CD8+ (DP) T-cells (%)	0.192	0.486	0.645	0.120	0.550
CD4+/CD8+ T-cell ratio	*0.066*	0.107	0.182	**0.001**	*0.062*

WBC, white blood cell count; ASC, antibody secreting cells; PBMC, peripheral blood mononuclear cells.

Bold value means *p*-value ≤ 0.05 and italic value means 0.05 < *p*-value ≤ 0.10.

A significant effect of time was found indicating a decrease in total serum IgM (*p* < 0.001), and a significant increase in *Borrelia*-specific IgG (*p* < 0.001). Furthermore, a significant effect of time was found for ‘total IgA ASC/500.000 PBMC’ (*p* = 0.003), with a significant increase at both D77 (*p* = 0.003) and D105 (*p* = 0.003). In contrast to what was found for IgA ASC numbers, a trend of decrease in the percentage of the well area covered with total IgA ASC was observed at D77 (*p* = 0.058). This indicates that numerous, but low IgA quantity producing ASC appeared following vaccination (Table 4).

**Table 4:** Summary of diet, time, age category or breed size effects for serum immunoglobulin, T-cell differentiation, ELISPOT and faecal data in both scFOS+ and placebo-fed dogs.

	Value	SE	*p*-value
Diet effect			
Total serum IgA (mg/dL) intercept^1^	286.5	24.5	**<0.001**
Total serum IgA (mg/dL) scFOS+ diet	−106.5	34.6	**0.006**
CD4 + CD8-T-cells (%) intercept	44.37	3.46	**<0.001**
CD4 + CD8-T-cells (%) scFOS+ diet	+8.31	4.91	0.106
CD4-CD8+ T-cells (%) intercept	37.09	3.48	**<0.001**
CD4-CD8+ T-cells (%) scFOS+ diet	−6.20	4.94	0.224
CD4-CD8-T-cells (%) intercept	16.36	1.40	**<0.001**
CD4-CD8-T-cells (%) scFOS+ diet	−1.48	1.99	0.464
CD4 + CD8 + T-cells (%) intercept	2.16	0.35	**<0.001**
CD4 + CD8+ T-cells (%) scFOS+ diet	−0.63	0.50	0.224
CD4+/CD8+ T-cell ratio intercept	1.52	0.52	*0.009*
CD4+/CD8+ T-cell ratio scFOS+ diet	0.79	0.74	0.296
Time effect			
Total serum IgM (mg/dL) intercept^2^	313.6	24.6	**<0.001**
Total serum IgM (mg/dL) D28	−150.6	33.3	**<0.001**
Total serum IgM (mg/dL) D105	−123.1	35.4	**0.001**
*Borrelia*-specific IgG (mg/dL) intercept	3.8	55.0	0.945
*Borrelia*-specific IgG (mg/dL) D77	+781.5	75.5	**<0.001**
*Borrelia*-specific IgG (mg/dL) D105	+446.3	76.8	**<0.001**
CD4 + CD8-T-cells (%) intercept	50.50	2.90	**<0.001**
CD4 + CD8-T-cells (%) D28	−4.81	2.21	**0.034**
CD4 + CD8-T-cells (%) D77	−2.90	2.34	0.220
CD4 + CD8-T-cells (%) D105	+0.15	2.39	0.950
Percentage of well area covered with total IgA ASC (%) intercept	35.5	7.8	**<0.001**
Percentage of well area covered with total IgA ASC (%) D77	−18.7	9.6	*0.058*
Percentage of well area covered with total IgA ASC (%) D105	−3.5	9.6	0.716
IgA ASC/500.000 PBMC intercept	1784	713	**0.018**
IgA ASC/500.000 PBMC D77	+1913	604	**0.003**
IgA ASC/500.000 PBMC D105	+1954	617	**0.003**
Faecal pH intercept	6.75	0.09	**<0.001**
Faecal pH D28	+0.23	0.11	**0.037**
Faecal pH D77	+0.14	0.11	0.213
Faecal pH D105	+0.22	0.13	*0.094*
Diet × time effect			
CD4 + CD8-T-cells (%) intercept	45.43	3.95	**<0.001**
CD4 + CD8-T-cells (%) scFOS+ diet	+10.28	5.64	*0.078*
CD4 + CD8-T-cells (%) scFOS+ diet D28	−2.47	4.52	0.588
CD4 + CD8-T-cells (%) scFOS+ diet D77	−3.32	4.82	0.494
CD4 + CD8-T-cells (%) scFOS+ diet D105	−1.80	4.90	0.716
CD4-CD8-T-cells (%) intercept	15.41	1.60	**<0.001**
CD4-CD8-T-cells (%) scFOS+ diet	−0.93	2.29	0.689
CD4-CD8-T-cells (%) scFOS+ diet D28	0.73	1.96	0.711
CD4-CD8-T-cells (%) scFOS+ diet D77	−4.61	2.09	**0.032**
CD4-CD8-T-cells (%) scFOS+ diet D105	0.80	2.12	0.709
CD4+/CD8+ T-cell ratio intercept	1.47	0.55	**0.014**
CD4+/CD8+ T-cell ratio scFOS+ diet	+1.25	0.79	0.124
CD4+/CD8+ T-cell ratio scFOS+ diet D28	−0.52	0.42	0.229
CD4+/CD8+ T-cell ratio scFOS+ diet D77	−1.18	0.45	**0.012**
CD4+/CD8+ T-cell ratio scFOS+ diet D105	−0.21	0.46	0.66
Breed size effect			
CD4+/CD8+ T-cell ratio intercept^3^	4.97	0.75	**<0.001**
CD4+/CD8+ T-cell ratio large breed	−3.42	0.93	**0.002**
CD4+/CD8+ T-cell ratio medium breed	−3.72	0.87	**<0.001**
CD4+/CD8+ T-cell ratio small breed	−3.34	0.99	**0.004**

SE, standard error; scFOS+: combo of pre- and postbiotics; D, day; ASC, antibody secreting cells; PBMC, peripheral blood mononuclear cells; FM, fresh matter.

1 Interpretation of data regarding diet effect: Dogs in the current study had an average total serum IgA level of 286.5 mg/dL (SE 24.5). Dogs fed the scFOS+ diet presented with an average decrease of 106.5 mg/dL regarding total serum IgA, resulting in an average level of 180 (=286.5–106.5) mg/dL total serum IgA for scFOS+ − fed dogs, and this finding was significant (*p* = 0.006).

2 Interpretation of data regarding evolution over time: Dogs in the current study had an average total serum IgM level of 313.6 mg/dL (SE 24.6) on day 0. On day 28 (D28), there was an average decrease in the concentration of total serum IgM of 150.6 mg/dL, and this finding was significant (*p* < 0.001). On day 105 (D105), dogs had an average total serum IgM level of 190.5 mg/dL (=313.6–123.1), and this finding was significantly different (*p* = 0.001) from the average overall total serum IgM level of 313.6 mg/dL.

3 Interpretation of data regarding breed size effect: Giant breed dogs in the current study had an average CD4+/CD8+ T-cell ratio of 4.97 (SE 0.75). Large breed dogs had an average decrease of 3.42 regarding CD4+/CD8+ T-cell ratio, and this finding was significant (*p* = 0.002). Medium breed dogs had an average decrease of 3.72 in CD4+/CD8+ T-cell ratio compared to the average ratio found in giant breed dogs, and this finding was significant (*p* < 0.001). Small breed dogs had an average CD4+/CD8+ T-cell ratio of 1.63 (=4.97–3.34), and this finding was significantly different (*p* = 0.004) from the average CD4+/CD8+ T-cell ratio of 4.97 in giant breed dogs.

Bold value means *p*-value ≤ 0.05 and italic value means 0.05 < *p*-value ≤ 0.10.

There were significant effects or trends found for diet (*p* = 0.022, *p* = 0.089 and *p* = 0.066), time (*p* = 0.099), and breed size (*p* = 0.008, *p* = 0.013 and *p* = 0.001) for the CD4 + CD8-T-cells (%), CD4-CD8+ T-cells (%) and CD4+/CD8+ T-cell ratio, respectively. In addition, a significant interaction between time and diet for CD4-CD8- T-cells (%; *p* = 0.042) and a trend for the CD4+/CD8+ T-cell ratio (*p* = 0.066) were seen, while no effect was observed for the age category (Tables 3, 4). Then, a multiple regression analysis was performed to test all those parameters by removing age category (Table 5). A significant effect of diet was found indicating an increase in CD4 + CD8- T-cells (%) (*p* = 0.011), a decrease in CD4-CD8+ T-cells (%) (*p* = 0.036), and a trend for a decrease in CD4 + CD8+ T-cells (%) (*p* = 0.059) in the scFOS+ group. As a result, the CD4+/CD8+ T-cell ratio increased (*p* = 0.009) in scFOS+ supplemented dogs. An effect of breed size was found, revealing a decrease in CD4 + CD8- T-cells (%), an increase in CD4-CD8+ T-cells (%) and a resultant decrease in the CD4+/CD8+ T-cell ratio (*p* = 0.011 *p* < 0.001) in small/medium/large versus giant breed dogs, respectively. Furthermore, a significant effect of time was seen at D77, with a decrease in the CD4+/CD8+ T-cell ratio (*p* = 0.011). A significant interaction between breed size and diet was observed as the CD4+/CD8+ T-cell ratios were significantly lower for small/medium/large breed dogs on the scFOS+ group versus giant breed dogs on the same diet (*p* < 0.001 for small/medium and large breed dogs; Table 6).

**Table 5:** Multiple regression analysis for T-cell subsets in dogs fed the scFOS+ and placebo diet.

	*Multiple regression*		
	Value	SE	*p*-value
CD4 + CD8-T-cells (%) intercept^1^	62.28	5.57	**<0.001**
CD4 + CD8-T-cells (%) large breed	−17.91	6.32	**0.011**
CD4 + CD8-T-cells (%) medium breed	−24.67	5.94	**<0.001**
CD4 + CD8-T-cells (%) small breed	−16.43	6.74	**0.026**
CD4 + CD8-T-cells (%) scFOS+ diet	+12.49	4.68	**0.011**
CD4 + CD8-T-cells (%) scFOS+ diet D28	−2.38	4.51	0.600
CD4 + CD8-T-cells (%) scFOS+ diet D77	−3.43	4.79	0.478
CD4 + CD8-T-cells (%) scFOS+ diet D105	−2.05	4.87	0.676
CD4-CD8+ T-cells (%) intercept	20.23	5.80	**0.002**
CD4-CD8+ T-cells (%) large breed	+16.37	6.61	**0.024**
CD4-CD8+ T-cells (%) medium breed	+23.51	6.22	**0.002**
CD4-CD8+ T-cells (%) small breed	+19.47	7.06	**0.014**
CD4-CD8+ T-cells (%) scFOS+ diet	−10.55	4.82	**0.036**
CD4-CD8+ T-cells (%) scFOS+ diet D28	+1.41	4.48	0.754
CD4-CD8+ T-cells (%) scFOS+ diet D77	+7.62	4.76	0.116
CD4-CD8+ T-cells (%) scFOS+ diet D105	+0.68	4.84	0.889
CD4-CD8-T-cells (%) intercept	16.04	2.96	**<0.001**
CD4-CD8-T-cells (%) large breed	−0.19	3.42	0.957
CD4-CD8-T-cells (%) medium breed	+0.03	3.22	0.992
CD4-CD8-T-cells (%) small breed	−3.20	3.66	0.394
CD4-CD8-T-cells (%) scFOS+ diet	−0.93	2.38	0.698
CD4-CD8-T-cells (%) scFOS+ diet D28	+0.73	1.96	0.712
CD4-CD8-T-cells (%) scFOS+ diet D77	−4.56	2.09	**0.034**
CD4-CD8-T-cells (%) scFOS+ diet D105	+0.84	2.13	0.693
CD4 + CD8+ T-cells (%) intercept	+1.37	0.64	**0.046**
CD4 + CD8+ T-cells (%) large breed	+1.72	0.74	**0.034**
CD4 + CD8+ T-cells (%) medium breed	+1.21	0.70	0.103
CD4 + CD8+ T-cells (%) small breed	+0.19	0.80	0.813
CD4 + CD8+ T-cells (%) scFOS+ diet	−1.01	0.51	*0.059*
CD4 + CD8+ T-cells (%) scFOS+ diet D28	+0.26	0.41	0.533
CD4 + CD8+ T-cells (%) scFOS+ diet D77	+0.42	0.44	0.346
CD4 + CD8+ T-cells (%) scFOS+ diet D105	+0.63	0.48	0.164
CD4+/CD8+ T-cell ratio intercept	4.54	0.73	**<0.001**
CD4+/CD8+ T-cell ratio large breed	−3.60	0.84	**<0.001**
CD4+/CD8+ T-cell ratio medium breed	−3.99	0.80	**<0.001**
CD4+/CD8+ T-cell ratio small breed	−3.53	0.91	**0.001**
CD4+/CD8+ T-cell ratio scFOS+ diet	+1.61	0.57	**0.009**
CD4 + CD8+ T-cells (%) scFOS+ diet D28	−0.51	0.42	0.234
CD4 + CD8+ T-cells (%) scFOS+ diet D77	−1.17	0.45	**0.011**
CD4 + CD8+ T-cells (%) scFOS+ diet D105	−0.21	0.46	0.643

Age category has been removed as it was not significant (see Table 3), while the interaction between time and diet was tested.

SE, standard error; D, day; scFOS+, combo of pre- and postbiotics.

1 Interpretation of data presented: Giant dogs fed the placebo diet in the current study had an average CD4 + CD8-T-cell percentage of 62.28% (SE 5.57) on day 0. Large breed dogs had an average decrease of 17.91% regarding CD4 + CD8-T-cell percentage, and this finding was significant (*p* = 0.011). Medium breed dogs had an average decrease of 24.67 in CD4 + CD8-T-cell percentage, and this finding was significant (*p* < 0.001). Dogs fed the scFOS+ diet presented with an average increase of 12.49% regarding CD4 + CD8-T-cell percentage, and this finding was significantly different (*p* = 0.011).

Bold value means *p*-value ≤ 0.05 and italic value means 0.05 < *p*-value ≤ 0.10.

**Table 6:** Multiple regression analysis for T-cell subsets in dogs fed the scFOS+ and placebo diet.

	Multiple regression			*p*-value of the model			
	Value	SE	*p*-value	Diet	Time	Breed size	Diet breed size
CD4 + CD8+ ratio intercept^1^	3.15	0.50	**<0.001**	**<0.001**	0.208	**<0.001**	**<0.001**
CD4 + CD8+ ratio D28	−0.37	0.22	*0.097*				
CD4 + CD8+ ratio D77	−0.31	0.23	0.195				
CD4 + CD8+ ratio D105	+0.02	0.24	0.941				
CD4 + CD8+ ratio large breed	−1.68	0.63	**0.017**				
CD4 + CD8+ ratio medium breed	−1.97	0.58	**0.005**				
CD4 + CD8+ ratio small breed	−1.70	0.67	**0.025**				
CD4 + CD8+ ratio scFOS+ diet	+5.88	0.82	**<0.001**				
CD4 + CD8+ ratio scFOS+ diet × large breed	−5.40	1.00	**<0.001**				
CD4 + CD8+ ratio scFOS+ diet × medium breed	−5.46	0.94	**<0.001**				
CD4 + CD8+ ratio scFOS+ diet × small breed	−5.19	1.06	**<0.001**				

Age category has been removed as it was not significant (see Table 3), while the interaction between breed size and diet was tested.

1 Interpretation of data presented: Giant dogs fed the placebo diet in the current study had an average CD4 + CD8+ T-cell ratio of 3.15 (SE 0.5) on day 0. Dogs fed the scFOS+ diet presented an average increase of + 5.88 regarding CD4 + CD8+ T-cell ratio, and this finding was significantly different (*p* < 0.001). A significant interaction between breed size and diet was observed as the CD4+/CD8+ T-cell ratios were significantly lower for small/medium/large breed dogs on the scFOS+ supplemented diet versus giant breed dogs on the same diet (*p* < 0.001 for small/medium and large breed dogs).

Bold value means *p*-value ≤ 0.05 and italic value means 0.05 < *p*-value ≤ 0.10.

## Discussion

The use of prebiotics and yeast derivatives may be most beneficial in animals with compromised immune systems, such as young weanling puppies, dogs under stress and geriatric dogs (11, 26). To the authors’ knowledge, only a handful of studies investigated the modulation of the immune system by prebiotics or yeast derivatives in senior dogs. Kroll et al. (27) investigated the effect of adding 0, 400 or 800 mg/kg of active fractions of mannoproteins derived from *Saccharomyces cerevisiae* yeast cell wall on both adult and old dogs for 28 days. Apart from reduced T-and B-cell counts with a lower T helper (CD4+) and a higher cytotoxic T-cell (CD8+) count with age, they showed that neutrophil activity and H_2_O_2_ production tended to increase after a lipopolysaccharide stimulation in the cells from dogs fed with 400 mg/kg mannoproteins compared to the control diet. In addition, the reaction ability of the cell-mediated immune response was modulated with 800 mg/kg yeast mannoproteins. Those results, even if mechanisms remain unclear at the time, suggest that yeast fractions may exert an effect on the immune system. Two other studies evaluated the effect of fiber on the faecal microbiota and the immune system of senior dogs (28, 29). Peixoto et al. (28) revealed that high resistant starch (1.46% vs. 0.21%) could affect the microbiota fermentation with no effect on gut mucosa parameters, whereas Maria et al. (29) showed an interaction between age and diet (fermentable fiber from beet pulp) on faecal IgA and an age effect on peripheral T-and B-cells, which were reduced in elderly animals. Finally, Grieshop et al. (30) specifically looked at the response of senior dogs supplemented with either 1% chicory (containing fructans), 1% MOS or a blend of 1% of each. The authors observed faecal microbiota changes together with a trend to increase blood neutrophil concentration with chicory alone and combined with MOS, while the lymphocyte concentration with MOS alone or combined with chicory decreased. These authors suggested that it may have been possible to detect more specific responses to the supplementation in case of an immune challenge. Likewise, Middelbos et al. (31) suggested that the full potential of a prebiotic supplementation to affect the immune system may not be visible unless the immune system is challenged. Therefore, the aim of the current study was to investigate whether dietary supplementation with scFOS combined with a yeast-based postbiotic could have a positive influence on immune parameters in healthy senior dogs during and after a vaccination protocol. Dogs had a good diet acceptance and maintained their weight throughout the study, with individual adjustments of food intake according to the metabolizable energy content of the experimental diets and the energy requirements of each animal. No significant difference was observed between groups on BCS and energy intake.

Faecal pH and scores were in a normal physiological range (32) suggesting that, as expected, our dogs were healthy. There was no diet nor ‘time × diet’ effect found for any of the faecal parameters included in the current study. A time effect for faecal pH with a significant increase at D28 and a trend for decreasing at D105 was detected. However, as the observed increases are rather small and the observed difference not larger than 0.5 units, the clinical relevance of these findings is probably insignificant. The current literature is inconsistent on the effect of pre−/postbiotic supplementation on faecal pH or scores. For example, Maria et al. (29) demonstrated that the faecal pH of senior dogs fed a diet containing 30% soybean meal (with fermentable oligosaccharides) was reduced compared with dogs fed a diet containing a nonfermentable sugarcane fiber. Furthermore, a study by Peixoto et al. (28) showed a decreased faecal pH in senior dogs fed resistant starch. Other studies performed on adult dogs, on the contrary, failed to show an effect on faecal pH (33, 34). In fact, several factors can contribute to explain the lack of effect: (1) the lack of sensitivity of the measure, (2) the dose of the pre-and postbiotics used, (3) the initial value of the pH with the control (depending also on the ingredients used in the diet), (4) and the high inter-individual variability of our cohort. In agreement with Maria et al. (29), the current did not find an effect of diet on the faecal score. In the study by Grieshop et al. (30), however, chicory + MOS increased the faecal score (1 = hard and dry, 5 = watery liquid), although these scores remained in a desirable range of 3–3.5, making difficult to give biological relevance of such results. Overall, our data suggest that scFOS+ was well-tolerated by and safe for an elderly dog population during the study duration.

No significant effects were found for complete blood count, serum cytokines nor acute phase proteins in the current study. It should however be mentioned that for SAA, 88% of the analyzed samples had values below the detection limit of 1 mg/L. SAA levels of 1.15 ± 2.53 mg/L are considered to be physiological values in healthy dogs (35). Furthermore, 98% of the analyzed samples had CRP values below the detection limit of 10 mg/L – a CRP level below 10 mg/L being considered normal in healthy dogs (36). These results strongly suggest that the dogs in the study, even though senior animals, were healthy, as it has been demonstrated that these two acute phase proteins are markers of systemic inflammation in dogs (37). As stated in the results section, the serum levels of the tested cytokines showed a huge variability, and the low number of dogs made it almost impossible to detect any significant differences. In humans, it is known that environmental factors, such as infections, and the genetic variation in a population are crucial for understanding the cytokine response to an infection or a vaccination. In our study, the genetic background of the dogs varied widely, and the enrolled animals were client-owned and thus lived in different environments. Those different factors may explain the wide variability and the lack of significant effects in the current study.

Regarding the serum IgA concentration, a significant decrease was observed in scFOS+ fed dogs. The clinical relevance of this finding is however unclear, as the serum IgA concentration in all dogs remained within the reference values of 19.0–390 mg/dL (38), except for 6 dogs exceeding this range on one or two occasions over the course of the study. The observed decrease in serum IgA is not necessarily to be considered negative and may indicate an immunoglobulin shift from mucosal to systemic immunity, possibly to aid in the immune response to Lyme vaccination, although no significant differences in vaccine-specific responses were observed. Glickman et al. (39) and HogenEsch et al. (40) observed elevated serum IgA concentrations in senior *vs* adult dogs. Therefore, the decrease in serum IgA observed in the scFOS+ supplemented dogs might indicate a countering effect on immunosenescence characteristics. Only one study (30) investigated the effect of prebiotics on serum immunoglobulin concentrations in senior dogs and revealed that supplementation of either MOS or chicory alone or in combination did not affect serum IgA, IgG or IgM concentrations compared to control dogs. Although such findings were also reported for scFOS and MOS supplementation in adult dogs (11, 26), the observed effect could possibly be caused by the *β*-glucans comprised in the postbiotic. Indeed, Stuyven et al. (41) reported a significant decrease in total serum and salivary IgA in adult dogs administered β-1,3/1,6-glucans from *Saccharomyces cerevisiae*, but this effect disappeared rapidly upon cessation of administration. They suggested that these changes in IgA reflected intestinal mucosal effects of the ß-glucans, for instance an increased local intestinal secretion of IgA, which was not assessed in the present study. More mechanistic studies are needed to better understand the effect of ß-glucans in the postbiotic used in the current study as it is well known that their molecular structure can impact the crosstalk with the epithelial and immune cells of the host (42).

Although there were no effects of diet, nor of ‘diet x time’ found for any of the other serum immunoglobulin parameters, the total IgM serum concentration significantly decreased over time. As IgM is the first antibody to appear in response to initial antigenic exposure (43), a decrease indicates that a stimulus causing acute stimulation of the immune system was removed. This finding could possibly be explained by the fact that all dogs were dewormed 3 days prior to the start of the study. Of note, serum IgM levels in all dogs remained within the reference values of 19.0–2,100 mg/dL (44). Furthermore, the highest titer of *Borrelia*-specific IgG antibodies was reached in the first weeks after vaccination, and values decreased from there on afterwards, demonstrating the seroconversion upon vaccination.

Although there was an effect of diet for total serum IgA, no diet effect was observed for *Borrelia-specific* IgA ASC. This could have been due to the systemic (subcutaneous) administration of the *Borrelia burgdorferi* vaccine, whereas the scFOS+ effect is probably the most pronounced at the gut mucosa. In addition, it is possible that the dose of prebiotics was too low to demonstrate more pronounced beneficial effects of scFOS+ supplementation under the vaccination conditions, as no effects were observed on vaccine-specific parameters in the current study. Although all dogs tested negative on the Canine Lyme Antibody Rapid Tests conducted prior to vaccination, we were able to detect *Borrelia* IgA ASC cells on day 28 in a very small percentage of dogs (blood samples collected immediately prior to vaccination). There was no effect of time (and therefore vaccination) for *Borrelia*-specific IgA ASC. It is possible that the sample collection time points post-vaccination were too late to be able to detect differences in circulating *Borrelia*-specific IgA ASC, as these ASC are known to be generated within about 6 to 8 days after antigen exposure, followed by a quick disappearance from blood in a period of days (45). However, in another study using the same research protocol in adult dogs (Wambacq et al., unpublished data), a significant effect of time was found for area % of the ELISPOT wells covered with *Borrelia*-specific IgA ASC post vaccination. In the current study, an effect of time was found for ‘IgA ASC/500.000 PBMC’ and ‘the percentage of well area covered with total IgA ASC’, indicating an overall increase in the IgA ASC numbers, but a decrease in the area percentage of the well covered at D77 as time progressed. Therefore, spots may have decreased in size as time progressed, with smaller spots in the ELISPOT assay indicating a lower IgA secretion rate (46). However, in case of high numbers of IgA ASC present in a single ELISPOT well, spot size might not the best parameter to be assessed. In such case, there will be competition for the nutrients present in the cell culture, with cells growing less and therefore secreting less IgA. The observed increased number of IgA ASC could not be attributed with certainty to the effect of vaccination, as there was no effect of time seen for *Borrelia* IgA ASC, making interpretation of this parameter difficult. A limitation of the study is the absence of a non-vaccinated control group throughout its entire duration. Although the first 28 days of the study allow for the assessment of dietary effects without the influence of vaccination, this period is not as long as the duration of the entire vaccination phase and subsequent follow-up. Furthermore, not all dogs entered the study on the same date, therefore the time frame of vaccination was not similar for all dogs.

Senior dogs generally have reduced blood CD4 + CD8- T-cells, increased CD4-CD8+ T-cell subsets and thus a reduced CD4+/CD8+ T-cell ratio (3, 40, 47–49). A significant effect of time - however not of age category - was seen in the current study, with a decrease in CD4 + CD8- T-cells (%) at D77, and a subsequent decrease in the CD4+/CD8+ T-cell ratio at D77. However, this effect was no longer visible after vaccination at D105, possibly due to the vaccine triggering the immune system. Considerable effort has been invested by pet food manufacturers in the past decades to investigate whether dietary modifications might counter the effects of T-cell immunosenescence. A study by Hall et al. (50) demonstrated that dogs consuming a diet containing 17 mg/kg of all rac-*α*-tocopheryl acetate had higher CD4+ to CD8+ T-cell ratios compared to diets containing higher vitamin E levels. In a study by Massimono et al. (51), supplementation of beta-carotene, a vitamin A precursor, in old dogs led to increased CD4 + CD8- T-cells. Therefore, there seems to be an optimal dose regarding dietary supplementation, with either too high or too low intakes resulting in suboptimal effects on the immune system. When focusing on the impact of prebiotics on altering the CD4+/CD8+ T-cell ratio in senior dogs, no effects have been reported when supplementing 400 or 800 mg active fractions of mannoproteins derived from *Saccharomyces cerevisiae* yeast cell wall per kg diet (27) or when comparing a diet containing 30% soybean meal to diets containing chicken byproduct meal with either 8.3% sugarcane fiber (nonfermentable fiber diet) or 10.4% beet pulp (fermentable fiber diet) (29). However, when comparing studies on the effect of prebiotics on the immune system, not only dosage of the supplement should be considered, but also the molecular structure (chain length, ramification, …) and the degree of purity of the supplement (e.g., the process by which the yeast cell wall was lysed), as certain compounds may mask the sugar components that bind to the respective immune cell receptors. Compared to placebo-fed dogs, scFOS+-supplemented dogs in the current study showed increased CD4+/CD8- T-cell numbers, decreased CD4-/CD8+ T-cells, and a resultant increased CD4+/CD8+ T-cell ratio, indicating the scFOS+ supplement countered certain characteristics of T-cell immunosenescence. Breed variation in the proportions of blood lymphocyte subsets has been demonstrated (51). However, this variation seemed unrelated to the breed size, as the highest (German Shepherds) and lowest (Dalmatians) CD4+/CD8+ T-cell ratios values were found within the category of large breed dogs. Giant breed dogs were not included in the aforementioned study. In the current study, giant breed dogs presented the highest number of CD4 + CD8- T-cells, the lowest CD4-CD8+ T-cell numbers and resultant highest CD4+/CD8+ T-cell ratios. It also appears that the effect of scFOS+ supplementation in countering certain characteristics of T-cell immunosenescence is largest in giant breeds dogs, as the interaction of diet x breed size was decreased for the large, medium and small breed dogs. Interestingly, blood from dogs fed scFOS+ had a lower percentage of mature CD4 + CD8+ double positive cells. Those cells have been described in different other animal species including humans, monkeys, and swine. They are also present in blood of dogs and share similarities with the cells described in humans and swine (53). Even if their specific functional roles (either suppressive or cytotoxic) are still poorly described and seem to differ according to the situation, in most of these species, these cells were defined to be part of the memory T cell pool, increasing with age and antigen contact (54). In agreement with that, the phenotype of canine CD4 + CD8+ T cells has been linked to activated effector/memory cells (55). Thus, a decrease in this pool of the T-cell population can also be viewed as a modulation of immunosenescence through the supplementation of scFOS+.

## Conclusion

In conclusion, the present study demonstrates that supplementation of diet with 1.1% scFOS+ to healthy senior dogs decreases total serum IgA concentrations, which might suggest a more local IgA response. In addition, the CD4+/CD8+ T-cell ratio was increased by scFOS+ supplementation during the study suggesting that it may counter certain characteristics of cellular immunosenescence in dogs. Further research is required to evaluate the effect of scFOS+ on the intestinal tissues namely the intestinal mucosal IgA production, B-and T-lymphocytes, microbiota composition and function and to validate the effects observed on immune parameters on a bigger cohort.

## Data Availability

The raw data supporting the conclusions of this article will be made available by the authors, without undue reservation.
